# *Punica granatum* and *Citrus* spp. Extract Mix Affects Spoilage Microorganisms Growth Rate in Vacuum-Packaged Cooked Sausages Made from Pork Meat, Emmer Wheat (*Triticum dicoccum* Schübler), Almond (*Prunus dulcis* Mill.) and Hazelnut (*Corylus avellana* L.)

**DOI:** 10.3390/foods8120664

**Published:** 2019-12-10

**Authors:** David Ranucci, Rossana Roila, Egon Andoni, Paolo Braconi, Raffaella Branciari

**Affiliations:** 1 Centro Interuniversitario per l’Ambiente (CIPLA), University of Perugia, Via Enrico dal Pozzo, 06123 Perugia, Italy; david.ranucci@unipg.it (D.R.); paolo.braconi@unipg.it (P.B.); 2 Department of Veterinary Medicine, University of Perugia, Via San Costanzo 4, 06126 Perugia, Italy; rossana.roila@studenti.unipg.it; 3 Faculty of Veterinary Medicine, Universiteti Bujqësor i Tiranës, Kodër Kamëz, SH1, 1000 Tiranë, Albania; eandoni@ubt.edu.al

**Keywords:** meat products, antioxidant, antimicrobials, shelf-life, plant extracts, pomegranate

## Abstract

Sausage made from pork meat, emmer wheat (*Triticum dicoccum* Schübler), almond (*Prunus dulcis* Mill.), and hazelnut (*Corylus avellana* L.) was integrated with a mix of *Punica granatum* and *Citrus* spp. extracts to evaluate the possible effects on the growth and oxidation of spoilage microorganisms. Two concentrations of the mix were added, respectively, during sausage-making, and the final products were compared with a control group, without the extract mix, during storage. The use of the mix, especially at 10 g/1000 g of the whole ingredients, delayed the pH drop and thiobarbituric acid-reactive substances (TBARs) value during storage. Total viable count, lactic acid bacteria and psychrotrophic microbial counts were also affected, as the extract mix lowered the maximum growth rate of the microbial population considered. The sensory analyses revealed an improvement in the shelf-life of 6 and 16 days, respectively, when 5‰ and 10‰ of the mix were used.

## 1. Introduction

The increased interest in food with healthy properties has led to a many studies on meat products in which meat is integrated or substituted at different levels with other ingredients, such as fibres, cereals and nuts [1,2,3,4]. The presence of cereals or products derived from them is reported in traditional meat products [5,6,7] and historically confirmed even form the Ancient Roman period [8]. Despite the presence of these meat products in the market, scarce information is available on their shelf-life and safety, especially if a high percentage of non-meat ingredients is added [8,9,10]. Moreover, the presence of non-meat ingredients may affect the microbial growth and oxidation and, consequently, the product shelf-life [8]. Additives are usually adopted to improve shelf-life and food safety, but consumers interested in healthier meat request products without synthetic additives (i.e., nitrites) or with natural substitutes that could increase aspects of both commercial stability and safety [4,11,12]. This study evaluated the effects of different concentrations of a commercial mix of pomegranate (*Punica granatum* L.) and *Citrus* spp. extracts (Naturmix WM^®^, MEC Import, Rome, Italy) on the growth of spoilage microorganisms and thereby the shelf-life of vacuum-packaged, cooked sausages made from pork meat, emmer wheat (*Triticum dicoccum* Schübler), almond (*Prunus dulcis* Mill.), and hazelnut (*Corylus avellana* L.), obtained from an Imperial Roman recipe. The extract mix was chosen to maintain a philological approach to this “historical” sausage, based on the evidence that both botanical species were present in the Mediterranean area during the Roman Age [13,14].

## 2. Materials and Methods

### 2.1. Sausage Production and Experimental Design

Sausages were produced in a local factory from shoulder pork meat (51% by weight of the ingredients), boiled emmer wheat (30%), equal parts of chopped peeled roasted almonds and hazelnuts (total of 15%), Squid Brand fish sauce (Thai Fishsauce Factory, Co., Ltd., Bangkok, Thailand) at 1%, salt and black pepper (3%). A control batch (C group) was produced without the mix addition when combining the ingredients, and two experimental groups were produced with 5 g/1000 g (MIX5 group) and 10 g/1000 g (MIX10 group), respectively, of the pomegranate and *Citrus* spp. mix. The meat was ground, as described in Ranucci et al. [8], and mixed with the remaining ingredients. Lamb small intestine casings were filled using a Piston stuffer (RL 15 IDRV, AMB Food Tech Ltd., Bologna, Italy) to obtain sausages of 25 g each (12 cm long by 1.5 cm in diameter) that were cooked in an oven (Self Cooking Centre, Rational AG Italia, Mestre, Italy) until reaching a core temperature of 72 °C for 1 min (registered with thermocouples inserted at the core of the product). The products were then cooled to 2 °C in a chiller (Tecnodom, Ristotecno Ltd., Gubbio, Italy), sealed under vacuum (A/PP 95, Seven Distribuzione, Città di Castello, Italy) in packs of five sausages and pasteurised on the surface inside the oven at 75 °C for 5 min (vacuum-sealed pre-cooked ready-to-eat meat product). The products were cooled again and then stored at 4 ± 1 °C.

Two batches were produced under the same conditions using the same recipe (three groups per batch). Five packs of products from each batch were immediately sampled for analytical determination after the final cool down (day 1 = T1), followed by samplings of the other packs after 7 days and every 15 days (five packs per batch at the following times: T2 = 7 days; T3 = 15 days; T4 = 30 days; T5 = 45 days; T6 = 60 days). From each pack, two sausages were randomly chosen for analytical determination, to obtain 10 sausages per group. Furthermore, 15 packs were collected for sensory analyses each sampling day.

### 2.2. Chemical and Physical—Chemical Determinations

The chemical composition and NaCl content of the products were determined at T1 by the following methods: moisture content by oven drying at 125 °C for 2 h (method 950.46) [15]; the Kjeldahl method for protein content (method 992.15) [15], using a nitrogen to protein conversion factor of 6.25; ether solvent extraction method for lipid content (method 960.30) [15]; high-performance anion-exchange chromatography with pulsed amperometric detection for carbohydrate content [16]; muffle furnace at 600 °C for the ash content (method 923.03) [15]; total soluble and insoluble dietary fibre (method 991.43) [17]; and the Volhard method for NaCl content (method 935.43) [14].

The thiobarbituric acid-reactive substances (TBARs) value was assayed according to Tarladgis et al. [18] at T1, T30 and T60, respectively, with measurement at 532 nm wavelength using a spectrophotometer (Ultrospec 2100 Pro, Amersham Pharmacia Biotech, Piscataway, NJ, USA), and the data was reported as milligrams of malondialdehyde per kilogram. The pH and water activity (*a_w_*) were determined every 15 days at the core of three sausages per group, using a pH meter equipped with an insertion probe (Crison 25, Crison, Barcelona, Spain) and a hygrometer (AquaLab Series 3 model TE, Decagon Devices, Inc., Pullman, WA, USA), respectively. The same measurements were performed in both batches of products.

### 2.3. Microbiological Analysis of Sausages

Sausages were aseptically sampled (25 g collected from the inner and outer parts of the products) in triplicate per group and homogenised in a stomacher (Stomacher 400 Circulator, Seward Ltd., Norfolk, UK) with 225 mL of sterile peptone water, followed by enumerating the microorganisms present.

Microbiological analyses of the total viable count (TVC), Enterobacteriaceae count and psychrotrophic microbial count (PMC) were performed according to ISO methods [19,20,21]; lactic acid bacteria (LAB) count on MRS agar incubated at 37 °C for 24 h under anaerobic conditions; and sulphite-reducing anaerobes on iron sulphite agar (Biolife, Milan, Italy) after anaerobic incubation at 37 °C for 48 h, were performed. Results of microbial analyses were expressed as log CFU/g.

*Salmonella* spp. were isolated [22], and *Listeria monocytogenes* presence was investigated [23].

### 2.4. Sensory Analysis

The samples of the three different sausages were tested at the various storage times by a panel of 51 untrained regular sausage consumers (27 female, 24 male), recruited among students and staff of the University of Perugia (Perugia, Italy), with ages ranging from 19 to 65 years [24]. The assessors provided their consent prior the tests, they did not receive any incentives for their participation, and the questionnaires were returned anonymously. No ethical approval was requested. The samples were prepared in a pre-heated oven at 200 °C for a time necessary to reach an internal temperature of 72 °C for 2 min (measured with a temperature probe). The samples were then placed in an isothermal container to maintain the temperature until serving, sliced in pieces of 2 cm length, assigned with a random three digit code and served. For each storage time, each judge, blind to the condition, evaluated the samples of each group (C, MIX5 and MIX10) three times. The overall acceptability of each product, which included the odour, texture and flavour (especially regarding rancid and fermented off-odours and off-flavours) attributes, was scored on a five-point hedonic scale, ranging from *Dislike very much* to *Like very much*. For the same samples, assessors were asked to evaluate acceptance or rejection of the sausages for the different storage periods, by answering the question: *‘Would you normally consume this product?’* with a *‘Yes’* or *‘No’* [25,26].

### 2.5. Statistical Analysis

Data were analysed by descriptive statistics (mean value and standard error of the mean), and an analysis of variance (ANOVA) model was defined using the GLM procedure in SAS version 2001 (SAS institute inc., Cary, NC, USA) considering the group (C, MIX5 and MIX10) and the time (T1, T2, T3, T4 and T5) as the fixed factor and including replicate (batch) as a random factor nested within the treatment and the time. Tukey’s post hoc test was used to compare the means with a significance level of *p* < 0.05. The batch effect was not significant for all the parameters tested (*p* > 0.05) and was not reported in the results.

The effects of the extract mix on the growth of the targeted microorganisms were evaluated through the DMFit function of ComBase online freeware, by fitting the experimental data obtained to the Baranyi–Roberts model, as automatically proposed by the software. For some of the growth curves elaborated by the DMFit, the lag phase was not defined. Therefore, in the absence of specific additional information concerning the actual initial physiological state of microbial populations, and to avoid bias in the growth kinetic evaluation, the lag phase parameter was not considered [27,28]. The results of fitting were analysed by one-way ANOVA (with the sausage group as fixed variable), and Tukey’s test (*p* < 0.05).

Survival analysis methodology was used to estimate the shelf-life by analysing the answers of the consumers to the question above using XLStat2015 software (Addinsoft, New York, NY, USA). The cut-offpoint was set by Weibull distribution, considering a 50% rejection probability by the assessors.

## 3. Results and Discussion

### 3.1. Chemical Composition and Physical—Chemical Determinations

The results of the chemical composition of the products are reported in Table 1. No difference in the registered values between groups was observed, and the data were consistent with those reported in Ranucci et al. [8] for a similar product.

The pH and *a_w_* values are reported in Table 2. The pH of all the products decreased as storage progressed, with lower values registered in group C than MIX10 at 45 days, and both MIX groups at 60 days. The pH decline during storage was registered in a similar product not subjected to post-packaging pasteurisation but at an early stage of 6–12 days [8]. The effect of LAB on the pH drop is reported in other fermented and cooked meat products [29,30,31,32], and these microorganisms that exert a favourable technological aspect in dry-cured sausages [33] could be considered spoilage bacteria in cooked meat products, especially if high levels of carbohydrate are present [9]. The increase in LAB concentration during storage is shown in Table 3. No differences were registered for *a_w_* values, which remained almost stable as storage progressed.

**Figure 1:**
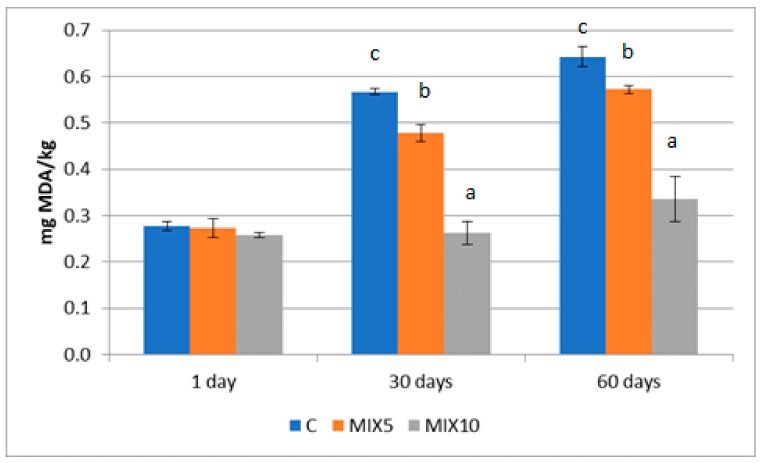
TBARs levels in the three groups of sausages. Different letters (a, b, c) are statistically different (*p* < 0.05); *n* = 10 samples per group of sausages. TBARs = thiobarbituric acid-reactive substances; MDA = malondihaldehyde.

The TBARs data are reported in Figure 1. The values registered were similar on day 1 (T1), then increased significantly at T3 only in C and MIX5 products, and at T5 for all the groups considered (*p* < 0.01). The differences between samples were registered from T3 onwards, with higher values in group C, followed by MIX5 and MIX10 products.

There is a general consensus in the literature that the antioxidant efficacy of pomegranate is effective in preventing food oxidation. The radical scavenging activity of pomegranate extracts is mainly due to the presence of polyphenols [34,35]. A dose-effect association with lipid oxidation is highlighted in cooked meat [36] in which the use of pomegranate extract delays the oxidation process during storage [37,38]. Other phenolic compounds are present in *Citrus* spp. extracts, in both free and bound forms that exert antioxidant activity both in vitro and in food systems [39,40]. Limited antioxidant activity may also be due to almonds and hazelnuts in the products, which contain phenolics and flavonoids, but in limited quantities compared with other nuts, like walnuts and pecans [41].

### 3.2. Microbiological Growth in the Sausages

Microorganism analyses (Table 3) revealed that all counts increased throughout storage, with higher loads for TVC, LAB and PMC in group C than in the treated groups after 60 days of storage. The presence of non-meat ingredients could be a valuable source of nutrients (carbohydrates) for bacteria to grow and proliferate in the products during storage, even at refrigeration temperatures [8]. These differences are noticed during storage of other meat products added with cereals, but do not always affect the microbial load at the end of the shelf-life [9]. Nonetheless, similar trends are reported in beef frankfurter-type sausages packaged under different modified atmosphere packaging and vacuum conditions without the addition of carbohydrate-containing ingredients [42]. The use of the blended extracts of pomegranate and *Citrus* spp. affected the microbial loads, as evidenced by the lower values in group MIX10 than MIX5 and C products from day 15. These data agree with the findings of Kannatt et al. [38], who noticed that the increased TVC in chicken products during storage was alleviated by incorporation of 1% and 5% pomegranate peel extracts, and with those of Firuzi et al. [43], who used different concentrations of pomegranate juice concentrate and rind powder extracts in frankfurters. This same trend was highlighted in raw meat added with pomegranate extracts [44,45]. The PMC was not affected by the pomegranate extracts in ground beef patties [46], but a reduction in the loads was registered in shrimp stored under refrigeration for 10 days and treated with different concentrations of pomegranate peel extracts [47]. Regarding *Citrus* spp. extracts, Mexis et al. [48] noticed a reduced growth rate in TVC, LAB, and pseudomonads in ground chicken meat. *Mortadella* meat products with incorporated citrus fibre, thyme essential oil and rosemary essential oil lowered the growth rate of both TVC and LAB during storage [48].

No literature data are yet available on the effects of *Citrus* spp. extracts alone on the microbial loads of cooked meat products.

**Figure 2:**
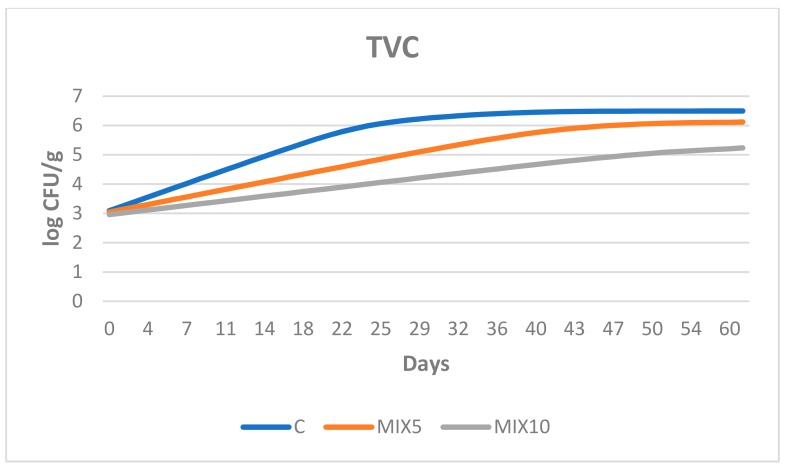

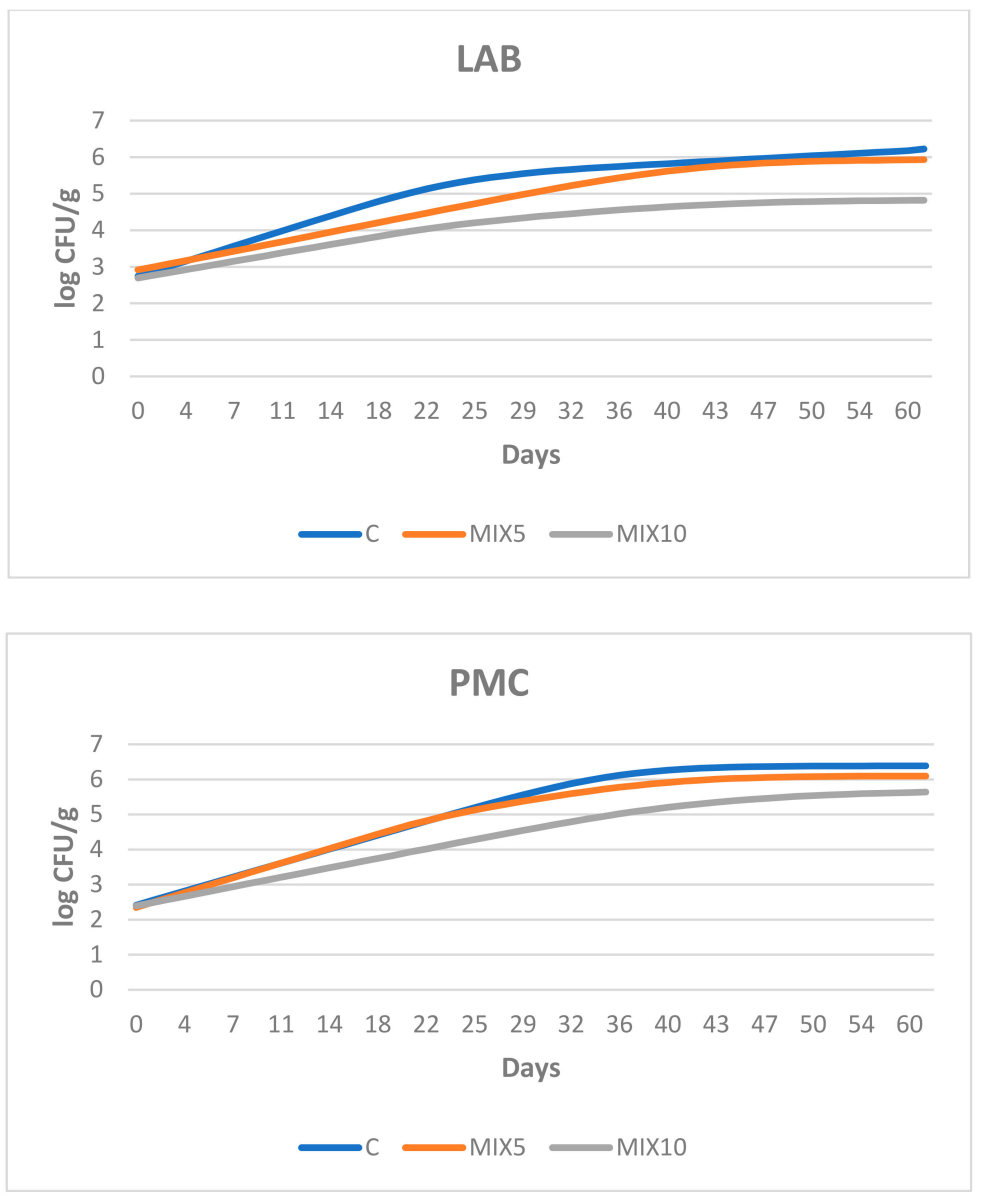
Predictive models of total viable count (TVC), lactic acid bacteria (LAB) and psychrotrophic microbial count (PMC) generated by the Baranyi–Roberts model in sausages with and without pomegranate and citrus mix. C = sausage made without *Punica granatum* and *Citrus* spp. extract mix; MIX5 = sausage made with 5‰ *P. granatum* and *Citrus* spp. extract mix; MIX10 = sausage made with 10‰ *P. granatum* and *Citrus* spp. extract mix.

The results from ComBase application to the microbial population are reported in Figure 2.

The Baranyi–Roberts no-lag model highlights differences in the *μmax* values (maximal growth rate) between groups for the microbial population considered. In particular, the values were 0.00539, 0.00299 and 0.00181 log CFU/h for TVC; 0.00483, 0.00302 and 0.00266 log CFU/h for LAB, and 0.00463, 0.00489 and 0.00313 log CFU/h for PMC in C, MIX5 and MIX10 products, respectively. The effect of the mix on the bacterial growth rate is highlighted by the difference found between the final values registered on day 60 (Table 3). The use of 10 g/1000 g of the mix decreased the maximum growth rate to a greater extent than the lower dose (5 g/1000 g).

Regarding the other microbiological analyses performed, the Enterobacteriaceae counts were always under the detection limit (1 log CFU/g) in the MIX10 group, while in the other groups, the counts increased throughout storage up to 1.33 and 3.35 log CFU/g in group MIX5 and C, respectively. The anti-microbial activity has already been reported for both pomegranate and *Citrus* spp. extracts [49,50,51], even inducing a decrease in the coliforms count in frozen beef sausages [52]. According to the values detected, a dose-dependent effect of the mix could not be established for the Enterobacteriaceae counts.

No sulphite-reducing anaerobes, such as *Salmonella* spp. and *L. monocytogenes*, were detected in the samples at any time considered. However, *Listeria* spp. were isolated from the products, as the pasteurisation protocol adopted was insufficient to eliminate the bacteria occasionally contaminating products after cooking [53], and both the pH and *a_w_* values of the products favoured *Listeria* spp. growth [54]. *Listeria* spp. were isolated from group C from T2 onwards, and group MIX5 at T5 and T6, but not in group MIX10. As reported by other authors, pomegranate extracts may exert activity against *Listeria* both in vitro [55] and in refrigerated pork meat [56] or delay *Listeria* growth when experimentally inoculated in cooked meat products [57]. The anti-listerial activity is related to the polyphenolic compounds, such as tannins [58], which cause bacterial protein precipitation, including disruption of the cell membrane [38,59] and enzyme inhibition [55]. Inhibition of *Listeria* spp. by *Citrus* spp. extracts are also reported [60], but some authors highlighted a limited in vitro activity for orange and lemon extracts [61].

### 3.3. Sensory Analyses for Shelf-Life

**Figure 3:**
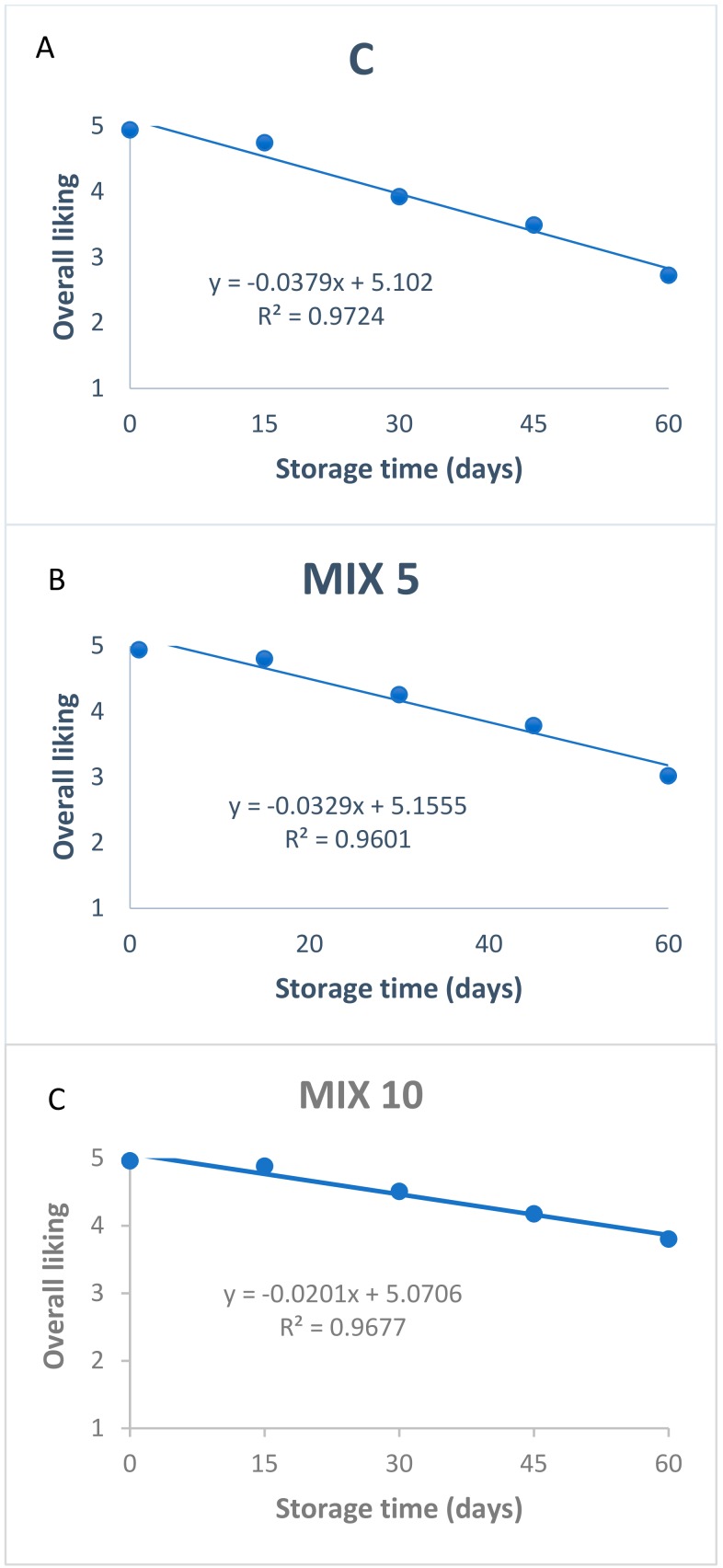
Assessors’ average overall liking scores as a function of storage time in sausages with and without pomegranate and *Citrus* spp. mix. (**A**) C = sausage made without *Punica granatum* and *Citrus* spp. extract mix; (**B**) MIX5 = sausage made with 5‰ *P. granatum* and *Citrus* spp. extract mix; (**C**) MIX10 = sausage made with 10‰ *P. granatum* and *Citrus* spp. extract mix.

The results of the sensory study for defining the shelf-life of the products are shown in Figure 3 and Table 4.

The higher stability of groups MIX10 and MIX5 than group C was supported by the sensory data. A linear regression analysis indeed was carried out considering assessors’ overall acceptability as a dependent variable and storage time as an explanatory variable (Figure 3). The approach used aimed to estimate sensory shelf-life as the time period during which consumers perceived the food item above the “neither like nor dislike” point (cut off 3.5), on a 5-point hedonic scale, as the acceptability limit for the product. The regression of the three products gave a good fit (R2 = 0.9724, 0.9601 and 0.9677, for C, MIX5 and MIX10 respectively), and using this regression, the control (group C) showed a decrease in assessors’ acceptability already at 42 days. Howsoever, at 60 days of storage the acceptability of MIX10 remained quite stable, scoring a value of 3.80 followed by MIX5, which was considered stable up to 50 days. During the storage the degradation processes were most evident in the C group, causing defects mainly in the taste as reported in the assessors’ comments (data not shown).

Shelf-life decisions based only on acceptability limit might be taken with caution, as they do not always reflect the consumer’s decision to accept or reject the product [62]. Indeed, survival analysis of food acceptability has been used to determine a proper shelf-life of the sausage.

The estimated shelf life of the products, set by the Weibull distribution of the sensory data (Table 4), revealed that 25% of the assessors reject the products at 30.815, 35.110 and 42.567 days for C, MIX5 and MIX10, respectively. Considering 50% probability of consumer rejection set as cut off point, the estimated shelf life was 44.487, 50.241 and 60.357 for C, MIX5 and MIX10, respectively. Thus, the shelf-life of such a product was improved up to 16 days when the concentration of the mix was increased (MIX10 vs. C). Shelf life estimated using survival analysis for a 50% consumer rejection is in line with the estimate determined by using an acceptability score of 3.5 as the failure criterion. This correspondence implies that during the products’ shelf lives, the consumers actually appreciate them.

A comparison of the shelf-life extension with other cooked sausage formulations found in the literature is difficult due to the differences in the composition of the products and the processing technology adopted. Authors referred to sausage with rice bran fibre added as having a shelf-life of fewer than 8 days, but no vacuum-packaging and surface pasteurisation were performed [63]. The shelf-life of sausages can be extended if packaged in modified atmosphere packaging and shrink packaging are used (54 and 45 days, respectively, in chicken frankfurters) [64] or stored under vacuum in refrigerating chambers (more than 8 weeks for cooked blood sausage) [65]. Even a post-packaging pasteurisation process could increase the shelf-life of cooked sausages [66].

## 4. Conclusions

The use of a mix of pomegranate and *Citrus* spp. extracts in a vacuum-sealed, post-packaged pasteurised cooked sausage made with meat and a high proportion of non-meat ingredients could be a valuable strategy to enhance the shelf-life by controlling both the microbial growth and oxidation during refrigerated storage. Food safety could also be improved, but further studies are needed to define the fate of specific foodborne pathogens in such sausages and to determine whether the single extract could exert specific effects against them and extend the products’ shelf-life.

## Figures and Tables

**Table 1:** Chemical composition of the sausages with and without pomegranate and citrus mix.

%	C	MIX5	MIX10	SEM
**Moisture**	51.08	51.21	51.15	0.022
**Protein**	14.55	14.96	14.56	0.080
**Lipid**	17.25	16.96	17.04	0.049
**Carbohydrate**	15.02	14.79	15.11	0.055
**Fibre**	7.62	7.56	7.64	0.014
**Ash**	2.10	2.07	2.14	0.012
**NaCl**	1.51	1.48	1.52	0.008

C = sausage made without *Punica granatum* and *Citrus* spp. extract mix; MIX5 = sausage made with 5‰ *P. granatum* and *Citrus* spp. extract mix; MIX10 = sausage made with 10‰ *P. granatum* and *Citrus* spp. extract mix. SEM = standard error of the mean.

**Table 2:** pH and water activity (*a_w_*) values of the sausages with and without pomegranate and citrus mix.

		*n*	Storage Time (Days)					SEM	*p-*Value		
			1	15	30	45	60		T	ST	T × ST
**pH**	**C**	10	6.61 w	6.23 x	6.18 x	6.08 ya	5.60 za	0.030	<0.001	<0.001	<0.001
	**MIX5**	10	6.64 w	6.20 x	6.17 x	6.12 yab	6.02 yb				
	**MIX10**	10	6.65 w	6.27 x	6.21 x	6.20 xb	6.14 yc				
***a_w_***	**C**	10	0.966	0.964	0.965	0.965	0.965	0.001	0.063	0.072	0.068
	**MIX5**	10	0.966	0.966	0.966	0.967	0.966				
	**MIX10**	10	0.970	0.966	0.967	0.966	0.965				

T = treatment (C, MIX5 and MIX10); ST = storage time; C = sausage made without *Punica granatum* and *Citrus* spp. extract mix; MIX5 = sausage made with 5‰ *P. granatum* and *Citrus* spp. extract mix; MIX10 = sausage made with 10‰ *P. granatum* and *Citrus* spp. extract mix. SEM = standard error of the mean. Values followed by different letters (a, b) in the same column for the same parameter are statistically different (*p* < 0.01); values followed by different letters (v, w, x, y, z) in the same row are statistically different (*p* < 0.01).

**Table 3:** Microbial counts of the sausages with and without pomegranate and citrus mix. Results are reported as log CFU/g.

		Storage Time (Days)						*p*-Value		
		1	15	30	45	60	SEM	T	ST	T × ST
**TVC**	**C**	3.05 v	5.19 wc	5.80 xc	6.15 yb	7.18 zc	0.068	<0.001	<0.001	<0.001
	**MIX5**	2.99 v	4.24 wb	5.11 xb	5.98 yb	6.11 yb				
	**MIX10**	2.85 v	3.83 wa	4.17 xa	4.87 ya	5.24 za				
***Lactobacillus* spp. count**	**C**	2.54 v	4.88 wb	5.12 wb	5.58 xb	6.85 yc	0.089	<0.001	<0.001	<0.001
	**MIX5**	2.86 v	4.11 wa	4.97 xb	5.82 yb	5.93 yb				
	**MIX10**	2.58 v	3.90 wa	4.15 wa	4.60 xa	5.04 ya				
**PMC**	**C**	2.26 v	4.47 wb	5.45 xc	5.97 yb	6.75 zc	0.101	<0.001	<0.001	<0.001
	**MIX5**	2.26 v	4.31 wb	5.16 xb	6.04 yb	6.27 yb				
	**MIX10**	2.20 v	3.89 wa	4.04 wa	4.99 xa	5.33 ya				

TVC = total viable count; PMC = psychrotrophic microbial count; T = treatment (C, MIX5 and MIX10); ST = storage time; C = sausage made without *Punica granatum* and *Citrus* spp. extract mix; MIX5 = sausage made with 5‰ *P. granatum* and *Citrus* spp. extract mix; MIX10 = sausage made with 10‰ *P. granatum* and *Citrus* spp. extract mix. SEM = standard error of the mean. Values followed by different letters (a, b) in the same column for the same parameter are statistically different (*p* < 0.01); values followed by different letters (v, w, x, y, z) in the same row are statistically different (*p* < 0.01). *n* = 10 samples per group.

**Table 4:** Estimated sensory shelf-life analyses of the sausages with and without pomegranate and *Citrus* spp. mix by survival analysis.

Percentage	C (Days)	MIX5 (Days)	MIX10 (Days)
1%	7.595	8.951	11.236
5%	15.000	17.390	21.464
10%	20.259	23.318	28.566
1^st^ Quartile 25%	30.815	35.110	42.567
Median 50%	44.487	50.241	60.357
3^rd^ Quartile 75%	59.419	66.637	79.480
90%	73.441	81.942	97.221
95%	81.971	91.217	107.930
99%	98.092	108.684	128.026

C = sausage made without *Punica granatum* and *Citrus* spp. extract mix; MIX5 = sausage made with 5‰ *P. granatum* and *Citrus* spp. extract mix; MIX10 = sausage made with 10‰ *P. granatum* and *Citrus* spp. extract mix.
